# Damned Drugs (Part II)

**Published:** 1954-10

**Authors:** G. E. F. Sutton


### DAMNED DRUGS

### Part II

### INAUGURAL PRESIDENTIAL ADDRESS

### Given at The Royal Fort, Bristol, on 8th October, 1952

BY

DR. G. E. F. SUTTON, M.C., M.D., F.R.C.P.

### Chloroform and the Pimlico Mystery

It is rare to find an ether addict, but occasionally one meets with an individual who is addicted to chloroform. I knew of a doctor who put himself to sleep every night by placing a handkerchief saturated in chloroform over his face. This is a very dangerous thing to do and it proved eventually to be fatal in his case. In the so-called Pimlico mystery it was claimed that chloroform was used with homicidal intent.

In 1875, Thomas Edwin Bartlett married a young Frenchwoman, Adelaine Blanche de la Tremoille, aged twenty, who was a native of Orleans. After their marriage Bartlett arranged for his wife to go to a boarding-school, so she lived with him only during the vacation. He then sent her to a convent school in Belgium, after which she settled down with her husband in London where he had several grocer's shops. Six years after their marriage Mrs. Bartlett gave birth to a stillborn child, and she was so upset by this event that she decided she would never have any more children. Four years later she and her husband made the acquaintance of a young Wesleyan minister, George Dyson, for whom they both conceived a great admiration. It was not long before they were on terms of what has been described as great social intimacy, and in 1885 Edwin Bartlett made a will leaving all he possessed to his wife and naming Mr. Dyson and his solicitor as his executors.

On December 10th of the same year Mr. Bartlett became seriously ill, but the astute practitioner who was called in to attend him thought that the state of the gums and the peculiar nature of the symptoms pointed to poisoning with mercury. He seemed to be gradually recovering, but nine days later he asked that a second doctor should be called in, " lest ", as he said, " if anything happened to me my friends should suspect that my wife was poisoning me ". Later it was said in court that this action was the result of some ill feeling which had formerly existed between his wife and his father.

The second practitioner was duly called in, and on December 26th, though still feeling weak, Mr. Bartlett had so far recovered as to be able to go for a drive.

On the following day Mrs. Bartlett asked Mr. Dyson (during one of his constant visits to the house) to procure for her a considerable quantity of chloroform. She told him, so the story goes, that her husband was afflicted with an internal complaint which at times caused paroxysms. She also expressed the opinion that he might die suddenly in one of these attacks. Mr. Dyson obtained three lots of chloroform—in all six ounces—from various chemists, stating that he required it for removing grease spots! Two days later he met Mrs. Bartlett on the embankment and handed it to her.

During his illness Mr. Bartlett slept on a camp-bed in the drawing-room and his wife slept on the sofa there. On December 31st he was quite fit again and at half-past ten on that evening Mrs. Bartlett told the maid that she required nothing else and retired with her husband for the night. At 4 in the morning she aroused the house, and it was found that her husband was dead in bed. She said that, after she had settled her husband for the night, she sat at the foot of the bed with her hand resting on his feet. After a time she dozed off and later awoke with a feeling of cramp and was horrified to find that her husband's feet were deathly cold. She tried to pour some brandy down his throat but found he was dead. She then aroused the household. The first person to enter the room was the landlord, who noticed a peculiar smell like "chloric ether".

The doctor was sent for at once, but could find nothing to account for death. A search was made, but the only bottle found was one that contained a few drops of chlorodyne. Autopsy showed that all the organs were healthy, but the stomach showed evidence of having contained a lot of chloroform.

The coroner's inquiry resulted in a verdict of wilful murder against Adelaide Bartlett and George Dyson and they were both arrested. But this was not the end. At the trial the Crown decided to offer no evidence against Dyson and he was duly called as a witness. Sir Edward Clark, who was said to be able to reduce any jury to tears, was called for the defence of Mrs. Bartlett.

The Rev. Mr. Dyson's examination occupied nearly the whole of the second day and he disclosed in some detail the form of intimacy between Mrs. Bartlett and himself. He admitted that he had purchased the chloroform and after hearing the result of the autopsy, had thrown the bottles away on Wandsworth Common while on his way to preach at Tooting. He said he had been in the habit of kissing Mrs. Bartlett and that her husband thoroughly approved of it. He had told him that he was becoming attached to his wife, but Mr. Bartlett had actually encouraged him and told him to continue the intimacy. The Rev. Mr. Dyson admitted that he did not tell Mr. Bartlett that he had procured the chloroform.

The doctor who had attended Mr. Bartlett then gave an account of an extraordinary statement which was made to him by Mrs. Bartlett after the death of her husband.

She said that she had been selected by Mr. Bartlett at the age of sixteen as a wife for companionship only. There was a sort of marriage compact in which they were to live together as loving friends and which precluded any carnal feeling. This strange and far from natural alliance was only terminated at the end of six years at Mrs. Bartlett's earnest and repeated entreaty that she should be allowed to become a wife and mother. The result of this late union was a stillborn child, and from that time onward there was no intercourse. Mrs. Bartlett further averred that her husband lavished on her an ultraplatonic affection. He encouraged her to pursue various studies, and liked to surround her with male admirers. She maintained that he encouraged the Rev. Mr. Dyson and herself to kiss in his presence, and not only did he enjoy it, but he gave her to understand that he had given her to him. Then, as her husband recovered from his illness, he expressed the wish that they should resume the ordinary relationship of man and wife, but we are not surprised to learn that she resented it and that she accordingly sought some means of frustrating his desire. It was thus that she asked her paramour to procure the chloroform.

On the night of her husband's death there had been some conversation of this sort, and when he was in bed she brought the bottle of chloroform and gave it to him, telling him that she intended to sprinkle some on his handkerchief and wave it in front of his face so that he might go peacefully to sleep. He looked at the bottle and placed it by the side of the camp-bed on which he was lying and, turning over on his side, apparently went to sleep. She also went to sleep sitting at the foot of the bed with her arm around his foot. Later, she heard him snoring, then woke again and found he was dead.

There can be little doubt that the recounting by the medical attendant of this strange history must have played no small part in the ultimate finding of the court. Certainly it enabled Sir Edward Marshall Hall to give full rein to his wonderful histrionic powers, and it is not difficult to visualize his listening to its unfolding with a relish that even this great dramatic criminal lawyer can rarely have known.

Dr. Stevenson stated that he found eleven and a quarter grains of pure chloroform in the stomach of the deceased, but judging by the time that had elapsed and the very volatile nature of the fluid, a large quantity must have been swallowed. He found no other poisons.

After deliberating nearly two hours the jury returned a verdict of "Not Guilty"!

Since the Pimlico poison case must remain among the unsolved mysteries, it is interesting to speculate on a few salient features which do not appear to have been sufficiently stressed.

It is well to recall, for instance, that when he was first called in to attend Mr. Bartlett on December 10th, the practitioner thought the state of his gums, together with the other symptoms, pointed to poisoning with mercury. Then why did the patient suddenly ask for another practitioner and give as his excuse for doing so: "If anything should happen to me my friends might suspect that my wife had been poisoning me"? Again, for what purpose did Mrs. Bartlett require chloroform? Why did she say that her husband had an internal complaint which caused paroxysms which she believed might end fatally? Yet Mr. Bartlett had been healthy in the past and the autopsy showed no evidence of internal disease. Again, in one breath we are told by a thinly veiled statement that there had probably been no marital relationship since the birth of her stillborn child, and that Mr. Bartlett's attitude towards his wife was so ultraplatonic as to be quite quixotic; yet, in the next breath, we are given to understand that, after his recovery, he wanted to resume normal marital life, much to the dismay of his wife.

Turning now to the Rev. Mr. Dyson, why did he buy chloroform from various chemists—six ounces of it—and why did he tell them that he wanted it for removing grease spots? (It is possible that even Mr. Bartlett might have taken some exception to being so described!) Why, when he heard of Mr. Bartlett's death, did he consider it necessary to dispose of the bottles on Wandsworth Common? We might also inquire why he purchased three lots of chloroform from three different chemists and then poured it all into one bottle.

We are informed of the peculiar attitude of Mr. Bartlett, of how he encouraged the intimacy of his wife and the Rev. Mr. Dyson, even approving of their kissing in his presence. Even assuming that he was somewhat quixotic, however, it is well to reflect that, had he been alive to do so, he might have thrown a very different and possibly more revealing light on the scene. He might, for instance, have smiled benignly at a friendly peck between his wife and the reverend gentleman who was his trusted friend. But even lovers are apt to embrace rather shyly in the presence of another, especially if that person happens to be the husband.

Be that as it may, we can, nevertheless, find some solace in the thought that had Mr. Bartlett died under similar circumstances to-day, an up-to-date counsel for the defence would have painted him with lurid colours as such a repulsive, perverted monster that the disgusted jury would have felt impelled to bring in a verdict of "Not Guilty".

We are not told whether Mrs. Bartlett and Mr. Dyson lived happily ever after, but we do know that she inherited all her husband's considerable possessions.

Some of you might think that the Scottish verdict of "Not Proven" would have been at least more appropriate than "Not Guiity" in this somewhat tangled case.

### Deep-dyed Death from Diphtheria

Both typhoid and cholera germs have been used for homicidal poisoning, but one of the most ingenious crimes on record was that in which diphtheria toxin was employed as the death-dealing agent.

In Petrograd in 1911 there lived a ne'er-do-well, Patrick O'Brien de Lacy, who claimed to be a lineal descendant of the Irish kings. Having met a lady of excellent family he easily persuaded her to divorce her husband and marry him. He subsequently spent all her money and then divorced her. Finally, having made the acquaintance of a Mlle Buturlin, he sought out a doctor, Panchenko, and conspired with him to poison the lady even before they were married.

Panchenko was a needy practitioner and O'Brien de Lacy offered him 10,000 roubles for the death of his brother-in-law, 50,000 roubles if he subsequently disposed of his father-in-law, and 500,000 roubles if he then dispatched his mother-in-law who was the wealthiest of the family.

In 1910 the younger Buturlin died at Petrograd after a week's illness. His father, old General Buturlin, who arrived from Vilna before the funeral, stopped the interment and demanded an autopsy. This being done it was decided that death was due to blood-poisoning and no further inquiry would have been made but for the fact that on that same day a man named Bobroff, who was employed by Panchenko as a tout to bring him patients, made a statement to the Chief of the Secret Police:

" A conscience is the only possession I can call my own, and it has driven me here to denounce my unique benefactress. She is my landlady, Mme Muraviova, who allows me a room in her flat and has been very kind to me. She is the mistress of Dr. Panchenko, with whom she has been hugger-muggering of late in suspicious ways. The door and walls being thin, I have heard snatches of conversation, which I have pieced together and I find that they point to Dr. Panchenko as the instrument of young Buturlin's death, and to O'Brien de Lacy as the employer of that instrument. The penniless Dr. Panchenko often journeyed to Vilna where O'Brien de Lacy resides and always returned with a fat purse and high hopes. Mme Muraviova, too, babbled about her improving prospects, saying she was shortly coming into 300,000 roubles. One day in April, Dr. Panchenko left for Kronstadt, where plague-stricken dogs are studied, and after his return he talked of little else. Soon afterwards young Buturlin, Panchenko and O'Brien de Lacy went on the spree together. The next thing I noticed was that Panchenko was weeping and sobbing. I entered the sitting-room and found him beside himself with excitement while his paramour was burning heaps of papers. She spoke first, saying to me that she had been scolding him for visiting a diphtheria patient without disinfecting himself. Then in an aside to Dr. Panchenko she asked: 'Did you do it properly ?' He answered: 'Well, I squirted two full doses although one would have been enough '. "

As a result of this story both Panchenko and O'Brien de Lacy were arrested. The former, after telling plausible stories, broke down and confessed the whole premeditated crime as follows:

" On May 16th I visited Buturlin and injected pure drug from a phial. I repeated the injection on the following day. Before visiting him in the evening of that day I broke the necks off the two drug phials in my lodgings so that nobody should notice it. Having emptied the contents I filled the phials with diphtheria poison by means of a paper funnel, plugged them with wadding and, putting them in my waistcoat pocket, set out for Buturlin's. Before starting I gulped down vodka for courage. I got to Buturlin's about 8 or 9 in the evening with trembling in my legs and throbbing waves of darkness filling my eyes and fitfully blotting out my sight. I had been wont to break off the phials in Buturlin's presence, first putting them in a handkerchief to avoid cutting my fingers. That is why he could not notice that this time the necks were already snapped off. I made two incisions in Buturlin's body, injecting each time the contents of one phial of the diphtheria poison. As soon as I had finished the business my face was ghastly and I quivered in every limb. I was in dread that Buturlin might discern my state. Pulling myself together and mastering my failing voice, I asked him whether it hurt. He answered: 'Not at all.' I then left for home and threw the phials in the street. The livelong night I could not close an eye. Conscience-ache racked me ruthlessly."

Despite this graphic story, it was subsequently revealed that Panchenko was a hardened criminal who, in addition to many other misdemeanours, had previously planned to poison several other people for money.

As a result of the trial, which lasted nearly three weeks, Panchenko was sentenced to fifteen years' penal servitude, and O'Brien de Lacy was sentenced to penal servitude for life. Panchenko's mistress, Muraviova, was acquitted.

It is interesting to recall that when epistaxis, sharp pains and vomiting ushered in the fatal agony, young Buturlin exclaimed: "Three months long they were at me to have the injections, but I refused, as though I had a presentiment of what was coming!"

### The Smethurst Case

The trial of Dr. Smethurst in 1859 for the murder of Miss Isabella Banks by poison was described by the Lord Chief Justice Pollock, who presided, as one of the most remarkable cases in his then long experience. Dr. Smethurst was at this time fifty-four years of age, though he gave his age as forty-eight. He was a man of small stature and insignificant mien, with a reddish-brown moustache. At the time he met Miss Banks he and his wife (who was twenty years older than himself) were living with her at a boarding-house in Bayswater, London. Their acquaintance ripened so quickly into a warm friendship that the landlady gave Miss Banks notice to quit. Soon afterwards Dr. Smethurst left his wife and joined her. A bigamous marriage took place at Battersea Church and the couple settled down at Richmond.

Miss Banks, aged forty-two, was a woman of some charm and considerable possessions. Apart from occasional bilious attacks she had always been healthy. Soon after her marriage she was taken ill and was attended by Dr. Smethurst who gave her all her food and medicines, and dealt with all the slops. She was complaining of soreness in the mouth and a burning sensation in the throat and intestines, with severe diarrhoea and vomiting, and as she was worse after some days it was decided to call in Dr. Julius, a well-known Richmond practitioner. Before long he began to suspect irritant poisoning and accordingly asked his partner, Dr. Bird, to see her. He, too, came to the same conclusion, but he did not say so until Dr. Julius had divulged his fears to him. During this time Dr. Smethurst continued in his apparently devoted ministrations to his new wife, and he was at some pains to keep her sister away, stating in his letters to her that their dear Bella was really too ill to see her, and as this was the doctor's word, she seems to have accepted it, after the manner of *some* relatives, more or less philosophically.

As Miss Banks's condition steadily deteriorated, Dr. Julius decided to call in Dr. Todd of King's College Hospital, who was one of the most eminent physicians of the day. (The temporary palsy which occasionally ensues after many epileptic fits, you will remember, was described by him and is hence known as Todd's Palsy.) Dr. Todd came to the same conclusion and had the excreta examined by the most distinguished toxicologist of the day, Dr. Alfred Swayne Taylor, Professor of Chemistry at Guy's Hospital. In one of the specimens he found arsenic, so Dr. Smethurst was promptly arrested, but was at once released by the magistrates. Miss Banks died next day and Smethurst was again arrested, this time on a charge of murder.

Two days before her death Dr. Smethurst visited a solicitor in Richmond, taking with him a draft will in his own handwriting, and requested the lawyer to come on the following day (which happened to be a Sunday) to execute it. This he did and Miss Banks, described in the document as a spinster, left everything she possessed—with the exception of a brooch—"to my sincere and beloved friend, Thomas Smethurst".

Autopsy revealed that she was from five to seven weeks pregnant. In the stomach there was a large patch of effused blood, but no signs of ulceration or acute inflammation. There was some inflammation at the commencement of the duodenum, the other parts of the intestine were slightly injected. In the lower three feet of the ileum, the mucous membrane was much thickened. That of the caecum was nearly destroyed by inflammation and ulceration. These appearances decreased along the colon and rectum. No arsenic whatever was found in the body, but antimony, in small quantities, was discovered in the caecum, the small intestine, one of the kidneys—the other not being examined—and in some blood taken from the heart.

The trial which took place at the Old Bailey was interrupted by the sudden severe illness of one of the jurymen and resumed a month later. At the onset there was a dramatic incident when Dr. Alfred Swayne Taylor admitted that he had made a mistake in saying that arsenic was present in one of the specimens of excreta. He stated that some arsenic was present as an impurity in the copper gauze which he had been using and this had been set free by some potassium chlorate which was present in the bottle in which the specimen was placed.

This resulted in the following letter being written to *The Times* by William Herapath, F.C.S., Professor of Chemistry at Bristol, and grandfather of the late Dr. C. E. K. Herapath, which is so justly scathing and so unanswerable that it must have had no small influence at the time:

"25th August, 1859.

"I consider that professional witnesses who give their opinions where the life or freedom of a man is at stake are as much upon their trial as the prisoner, and that it is the duty of those who are well acquainted with the particular science they profess to correct any error that might deceive the jury or influence the fate of the prisoner. With these views, from my experience in cases of poisoning, I think I ought to remark upon the chemical evidence adduced in the case of Dr. Smethurst. I find in *The Times* of 21st May that Dr. Taylor deposed that in Bottle No. 21 he found it half-full of a clear fluid, that he tried Reinsch's process on it, and found that there were seven grains of chlorate of potash in an ounce of it, which was 1·6 per cent, and that there was a grain of arsenic to every ounce; he further said that it was of the sort we call white arsenic, and that he had previously tried the tests (materials) and found them pure. Here was evidence of the most positive and distinct kind; not only was arsenic found, but the weight estimated and its nature (white) ascertained. Upon the trial this was stated to be a mistake; that, although the former evidence was given so boldly and so particularly, it was now found that there was no arsenic in it, but that the arsenic found was in the copper, although the copper had, with the other materials, been previously proved to be pure. The witness went on to say that he had at first operated upon an evacuation, and found arsenic in that, but admitted that he had used the same copper for many years; consequently all proof of the presence of the poison either in the body or in the evacuation, or in the bottle in the possession of the prisoner, was destroyed by the witness himself, and the jury must rest upon the symptoms and physiological appearances in the opinions of the medical witnesses called, which are about equal for and against the prisoner. But the mischief does not end here, for if the same impure copper 'has been used for twenty years', and evidence given upon it, what shall be said of the justice of the convictions and executions which have taken place during those years upon Dr. Taylor's evidence?

"But was the arsenic said to be found in Bottle No. 21 really in the copper used to prove its presence? Could the copper wire gauze dissolved by seven grains of chlorate of potash and its associated hydrochloric acid deposit one grain of arsenic? In the face of all England I say it could not; the hundredth part of a grain of arsenic in that quantity of copper would render it so brittle that it could not be drawn into wire at all, much less into fine wire fit for gauze; the fact is, the whole set of operations were a bungle. Reinsch's process is not applicable where nitrates or chlorates are present.

"Next, where his admirable process is resorted to, the mere discoloration of the copper does not prove the presence of arsenic; it only proves that one or more of the inferior metals—arsenic, antimony, tin, lead, bismuth, mercury, etc., are present.

"To individualize arsenic among these four more experiments are necessary. The first is sublimation in a stream of air, when crystalline arsenious acid is produced from the black deposit. Next, that sublimate must be dissolved in water and tested by three methods: first, ammonia, sulphate of copper; next, ammonia, nitrate of silver; and, thirdly, sulphuretted hydrogen.

"Thus, one separation and four proofs produce a body of evidence which it is impossible to gainsay, and these five proofs should be brought into Court so as to be examined by those who are competent to recognize them; and I here warn juries that no evidence short of tangible production of the poison and its tests ought to be for one moment attended to; any deviation from this rule will convert a convicted criminal into a martyr, and deprive trial by jury of the infallibility which it ought to possess; and, lest it might be imagined that it may not be possible to secure enough of the poison to make the five proofs, I should say that the one-thousandth part of a grain is quite sufficient."

Then began a conflict of medical evidence the like of which has perhaps never been equalled even to this day. On the one hand there were a number of physicians, including the two practitioners and Dr. Tod, who were convinced that the picture was that of an irritant poison, and on the other hand there were a number of eminent physicians who were equally certain that the symptoms and the autopsy findings were typical of dysentery. Finally, the gynaecologists were quite sure that the patient's symptoms had all been caused by the toxaemia of pregnancy, and they quoted what appeared to be several similar cases in support of their opinion.

Mr. Sargent Parry for the Defence promptly asked Dr. Julius:

"Are you a doctor of medicine?"

"Yes."

"Is yours a London degree?"

"Yes."

"What degree is it?"

"It is the Archbishop of Canterbury's degree."

"What! Can he make a doctor of medicine?"

The Lord Chief Justice: "Yes, and he can also make a Master of Arts."

Mr. Sargent Parry: "Did you take your degree as a matter of course?"

Dr. Julius: "Oh dear, no! It is a very uncommon thing. I had to get a certificate from two members of the College of Physicians stating that they had known me for a length of time and that I was a proper person to have the degree."

Mr. Sargent Parry: "And having that certificate you got the degree?"

"Yes, but it only entitles me to call myself doctor."

The Lord Chief Justice: "But you are a member of the College of Surgeons and a member of the Society of Apothecaries?"

"Yes."

The power to grant medical degrees was a remnant of papal authority which was reserved to the Archbishop of Canterbury by statute passed in 1533 in the reign of Henry VIII. The Medical Act of 1857 abolished any qualification to practise with these degrees unless they were granted prior to the passing of the Act. The Archbishop does not now use his power to confer these empty honours.

In his charge to the jury the Lord Chief Justice left little doubt that the evidence pointed strongly towards Dr. Smethurst's guilt. Apart from the almost immediate illness of Miss Banks following the bigamous marriage, he pointed out that, while Smethurst had kept her sister from the bed of her nearest relative, giving as an excuse the critical state of her health, he yet insisted on the attorney coming in at that moment and against his wishes on a Sunday, to legalize the will which he himself had drawn up in his own handwriting. In that will Isabella Banks was described as a spinster and she appears to have made some feeble protest at this and then signed the will.

The Lord Chief Justice also pointed out the manner in which Smethurst had secluded Miss Banks from everyone, and that on one of the rare occasions when the sister was allowed to see her, she brought some soup which he would not allow to be given, and when she wanted to make her some arrowroot he would not allow her to do so. Again, whereas Miss Banks was vomiting after everything that Smethurst gave her, when Dr. Bird gave her some food and brandy after Smethurst had been arrested the first time, she kept it down. Further, not only was no nurse employed throughout, even for the offices of the sickroom, but when, after his discharge from prison, it was suggested he should employ one, Smethurst refused, saying: "Let Dr. Julius provide these things at his own expense. I am told to interfere no more." Yet he already had £71 of Miss Banks's money in his possession at that time.

It was further pointed out that at the time of his arrest there was found in Smethurst's pocket a letter to his seventy-four-year-old wife, as follows:

" My dearest Mary,
I have not been able to leave for town as I expected in consequence of my medical aid being required in a case of illness. I shall, however, see you as soon as possible, and should any unforseen event prevent my leaving for town before the 11th [the 11th was the day of the month on which payment from Miss Banks's invested money was procured] I will send you a cheque for Smith's money and extras. I will send you £5. I am quite well and sincerely hope you are the same and that I shall find you so when I see you which I trust will not be long first. Present my kind regards to the Smiths and all friends in the house. I heard from James the other day; he said he had called upon you but you had gone out for a walk."

The Lord Chief Justice commented on this to the jury as follows:
" You are to judge whether this is the letter written by a man to his wife whom he has abandoned for ever, or whether it is written by a man who means to go back to her and thinks that the business on which he left her is drawing to a close."

The Lord Chief Justice stated that it may be that no poison is traced to the possession of the accused, and it may be that none is found in the body, and yet it may be the easiest thing in the world to put a case where no sensible man could doubt that the accused had possessed the poison, had used it, and that the deceased had died of it. He added that this is no doubt a case of circumstantial evidence, and it creates a duty on the part of the jury to consider first—" Do you believe the evidence? Do you believe this fact, that fact? The next? And so on; if you do, what do they prove? I can understand it being said: would you find the man guilty for the first? No, certainly not. Would you for the second? No. Would you for the third? No. Or the fourth? No. But what would you do if they were all together? It is really like this—there is a scale with a hundred-pound weight in one of the scales. Will five pounds lift it? No. Will eighty? No. But what will they do altogether? Why, outweigh it abundantly, lift it without any difficulty, and that is really, I think, a just explanation of what may be called the theory of circumstantial evidence. You estimate the value of a fact and it is perfectly indifferent—but coupled with another fact it may be exceedingly important."

The jury returned a verdict of " Guilty ", but, nothing daunted, when called up for judgment, Smethurst launched upon a long harangue on why he was innocent, during which he defamed Dr. Julius in every way possible. Then, when the Lord Chief Justice pronounced sentence of death, he exclaimed: " I declare Dr. Julius to be my murderer. I am innocent before God! "

If the trial had been eventful and even startling at times, it was but a wave in a pond compared with the storm which it evoked. You have already heard William Herapath's scathing denunciation, but both in the lay and medical press there were many more equally scathing, both with regard to the trial and the conviction.

The Secretary of State was petitioned for pardon in a memorial signed by thirty practitioners of London and in yet another signed by twenty-eight members of the bar. Smethurst himself was most active, not only in writing to the *Lancet* and *The Times*, but in petitioning the Secretary of State. His real wife also petitioned on his behalf and she averred that, apart from his one lapse, he had been throughout a loving and devoted husband. His brother, who was a chemist, also wrote to *The Times* on his behalf. Eventually the Secretary of State became so worried with it all that he took the unprecedented action of submitting all the case-notes to the leading surgeon of the day, Sir Benjamin Brodie. In his reply to the Home Secretary, Sir Benjamin made it clear that although the facts were full of suspicion against Smethurst, there was not absolute and complete evidence of his guilt.

The Home Secretary, therefore, advised Her Majesty to grant a free pardon, stating that the necessity of doing so in this case arose from the imperfection of medical science and from fallibility of judgment, in an obscure malady, even of skilful and experienced medical practitioners. This statement, which was as true then as now, created great offence among the medical profession of the day.

After his release Smethurst was promptly arrested, tried and convicted for bigamy. He was sentenced to one year's hard labour. During this trial it was disclosed that his first wife had committed bigamy when she married him.

Smethurst was no sooner released from prison when he took immediate steps to prove the will of Isabella Banks in his favour. Needless to say, many scathing comments were made by counsel at this hearing, but they fell off Smethurst like water off a duck's back, and in the end he proved the will and retired with the very comfortable income.

In his letter to the Home Secretary, Sir Benjamin Brodie made this comment: "There is another circumstance which, trivial as it would appear to be, would, in some degree, have influenced my opinion if I had been on the jury, namely, the proposal which Smethurst made to test the strength of the prussic acid which was to be administered to the patient by dipping bread in it to be given to the sparrows and which seems to show that the subject of poisoning was not altogether new to him, and that he must have been in the habit of dabbling in experiments with them."

While I have been engrossed in my grim discourse, many of you, as thoughtful men and women, will have asked yourself the question: "What is the underlying mental make-up of the poisoner, and how does it differ from that of the normal individual?" In his foreword to the book by L. A. Parry, M.D., F.R.C.S., on the Trial of Dr. Smethurst, Mr. John Flowers, K.C., writes these words:

"It has been truly said that most of the interest and part of the terror of great crime are due, not to what is abnormal, but to what is normal in it. Revenge, avarice and passion are often dominating influences in the life of normal persons, and yet one or other of such influences is usually the motive for murder. The normal person in this world has, therefore, something, however slight, in common with the criminal, and naturally looks with interest and amazement at the tragic depths to which a man may sometimes fall under the influence of motives which are common to his fellow men. If this is not so, it is a little difficult to understand the extraordinary outbursts of feeling in favour of a person who is suspected of having committed a murder. For this reason he sometimes becomes a popular hero, quite apart from the merits or demerits of the case which is made against him, and his acquital, which may often mean no more than a case of not proven, is made the occasion of misplaced and ridiculous hero worship."

We might well add that "since all the world's queer except thee and me, and even thee's a bit queer", "there, but for the Grace of God, goes John Bunyan", preceded by all and sundry. But the problem of the psyche and its motivation is not a simple one. The late Justice Darling, when asked the difference between the mind of the murderer and that of the normal individual, stated that the murderer was a sublime egoist, so that everything had to be sacrificed to the attainment of his selfish ends. The learned judge did not consider that he differed materially in other ways from normal individuals. Certainly there is much egocentricity in normal people, and it must be remembered that in the struggle for existence, through countless years, no one would be here now but for a high degree of this characteristic in his progenitors. It has even been said that it is normal for people to wish that their chief competitors in life were dead, and for the young man to wish a pox or death upon his rival in love. Without entering more deeply into this interesting question, which clearly must involve environmental influences as well as the question of faith, I will only add that, in my opinion, quite apart from his egotism, the murderer is particularly lacking in what the psychologists term "affect tone". Putting it even more simply, he is completely devoid of that warm human feeling which, with all its faults, is shared, in not such varying degree, by the common mass of humanity. This lack must be specially true of the poisoner, who, nevertheless, possesses a stern fortitude which is in keeping with his grim resolve.

Finally, you will say that I have portrayed for you a seamy and sordid picture of

" the lusts that lure us on, the hates that hound us; our red rags in this patchwork quilt of life ". But if " all the world's a stage and all the men and women merely players ", there must be few who have the opportunity of playing a more important part in the act of life than the doctor, whether for good or ill. Who better than he can feel that though there are treacherous undercurrents on the one hand, on the other " there are waters blown by changing winds to laughter ".

And of those who have been prematurely removed from this transitory sphere by the fell hands of the poisoner, at least we can say:

> Secure from worldly chances and mishaps
> Here lurks no treason, here no envy swells.
> Here grow no damned drugs,
> Here are no storms, no noise,
> But silence and eternal sleep.

So let us leave them, perhaps reflecting that, after all, " the surest poison is Time ".

